# Intraluminal Blockade of Cell-Surface CD74 and Glucose Regulated Protein 78 Prevents Substance P-Induced Bladder Inflammatory Changes in the Rat

**DOI:** 10.1371/journal.pone.0005835

**Published:** 2009-06-08

**Authors:** Pedro L. Vera, Xihai Wang, Richard J. Bucala, Katherine L. Meyer-Siegler

**Affiliations:** 1 The Bay Pines VA Healthcare System, Research & Development, Bay Pines, Florida, United States of America; 2 Department of Surgery, Division of Urology, University of South Florida, College of Medicine, Tampa, Florida, United States of America; 3 Department of Internal Medicine, Yale University School of Medicine, New Haven, Connecticut, United States of America; 4 Department of Molecular Medicine, University of South Florida, College of Medicine, Tampa, Florida, United States of America; Instituto Oswaldo Cruz and FIOCRUZ, Brazil

## Abstract

**Background:**

Macrophage migration inhibitory factor (MIF) is a pro-inflammatory cytokine constitutively expressed by urothelial cells. During inflammatory stimuli, MIF is released into the lumen complexed to other proteins and these complexes can bind to urothelial cell-surface receptors to activate signaling pathways. Since MIF is complexed to α1-inhibitor III (A1-I3; a member of the α2-macroglubulin family) and glucose regulated protein 78 (GRP78) is a receptor for A1-I3 the goals of this study were to determine if substance P elicits urothelial cell-surface expression of GRP78 and to assess the functional role of CD74 (receptor for MIF) or GRP78 in substance P-induced bladder inflammatory changes.

**Methodology/Principal Findings:**

Anesthetized male Sprague-Dawley rats received either saline or substance P (s.c.), bladders were collected 1 hour after treatment and processed for histology or protein/mRNA. The expression of GRP78 at urothelial cell-surface was determined by performing in vivo biotinylation of urothelial cell-surface proteins. Finally, in order to determine the effects of receptor blockade on substance P-induced MIF release and inflammatory changes, rats received either intraluminal antibodies to CD74, GRP78, both, or non-specific IgG (as a control).

GRP78 and MIF immunostaining was simultaneously visualized in umbrella cells only after substance P treatment. Immunoprecipitation studies showed GRP78-MIF complexes increased after substance P while in vivo biotinylation confirmed substance P-induced GRP78 cell-surface expression in urothelial cells. Intraluminal blockade of CD74 and/or GRP78 prevented substance P-induced changes, including bladder edema, intraluminal MIF release by urothelial cells and production of inflammatory cytokines by urothelial cells.

**Conclusions/Significance:**

GRP78 is expressed on the surface of urothelial cells after substance P treatment where it can bind MIF complexes. Blocking CD74 (receptor for MIF) and/or GRP78 prevented substance P-induced inflammatory changes in bladder and urothelium, indicating that these urothelial receptors are effective targets for disrupting MIF-mediated bladder inflammation.

## Introduction

Macrophage migration inhibitory factor (MIF), a pleiotropic pro-inflammatory cytokine[1] found in many different cells (including urothelium), is associated with experimental cystitis[2]–[7] and urinary tract infection in humans[8]. MIF is released (complexed to other proteins[8], [9]) into the lumen during cystitis and this process depends on activation of bladder afferent and efferent nerves[7]. The source of released MIF is unknown, however, since MIF is constitutively expressed in the basal and intermediate layers of the urothelium[10], and MIF mRNA is upregulated in the urothelium during inflammation[11], it is likely that the urothelium is either responsible for MIF release during cystitis or contributes a significant portion of the MIF released. Examining changes in urothelial MIF content and also changes in inflammatory markers in the urothelium would answer questions about the source of released MIF during cystitis and would also support an active role for the urothelium during inflammation. This is an important question and would indicate that the urothelium is an active respondent to bladder injury and participates in inflammatory signal transduction[12].

Once released into the lumen, MIF and/or MIF-complexes may bind cell-surface receptors (e.g. CD74[13]; CXCR4[14], [15]) to activate pro-inflammatory signal transduction cascades and thus maintain or enhance bladder inflammation. In fact, both CD74 and CXCR4 have been shown to be upregulated in the bladder during cystitis [4], [6], [15]. Moreover, using an in vivo biotinylation procedure, we recently showed that substance P (SP) induces cell-surface expression of CD74 in urothelial cells which allows MIF-CD74 binding[11]. Thus, during cystitis not only is there MIF released into the lumen but also expression of receptors on the surface of urothelial cells that bind to MIF to maintain or promote bladder inflammation. Accordingly, blocking MIF-receptor interactions may be a means to pharmacologically disrupt bladder inflammation.

We showed in the rat that MIF is released complexed to α1-inhibitor III (A1-I3; an acute phase protein in the α2 macroglobulin family)[9] and we also showed that MIF and A1-I3 immunostaining are observed simultaneously in umbrella cells after substance P (SP) treatment[16]. Since glucose-regulated protein 78 (GRP78) is a receptor for A1-I3[17], we hypothesized that SP elicits cell-surface GRP78 expression in umbrella cells with the ability to bind MIF-A1-I3 complexes. GRP78 (an endoplasmic reticulum stress protein activated as part of the unfolded protein response[18]) localizes to the cell-surface and mediates signal transduction[17], [19], although the role of GRP78 in bladder inflammation is not known. Therefore, in the present study we investigated the effects of SP on GRP78 expression in urothelium and the functional role of GRP78 or CD74 (receptor for MIF recently shown by us to be expressed on the urothelial cell surface after SP treatment[11]) on SP-induced bladder inflammation in rats.

In these experiments we aimed to: 1) examine SP-induced GRP78 and MIF immunostaining in the urothelium; 2) examine SP-induced MIF-GRP78 complexes in the bladder using co-immunoprecipitation; 3) examine SP-induced urothelial cell-surface expression of GRP78 using in vivo biotinylation of urothelial cell-surface proteins and GRP78 immunoblotting; 4) test effects of intraluminal blockade with antibodies to CD74 and GRP78 on SP-induced bladder inflammation, urothelial MIF release and inflammatory cytokine expression.

## Results

### Substance P-induced GRP78 and MIF immunostaining in umbrella cells

**Figure 1:**
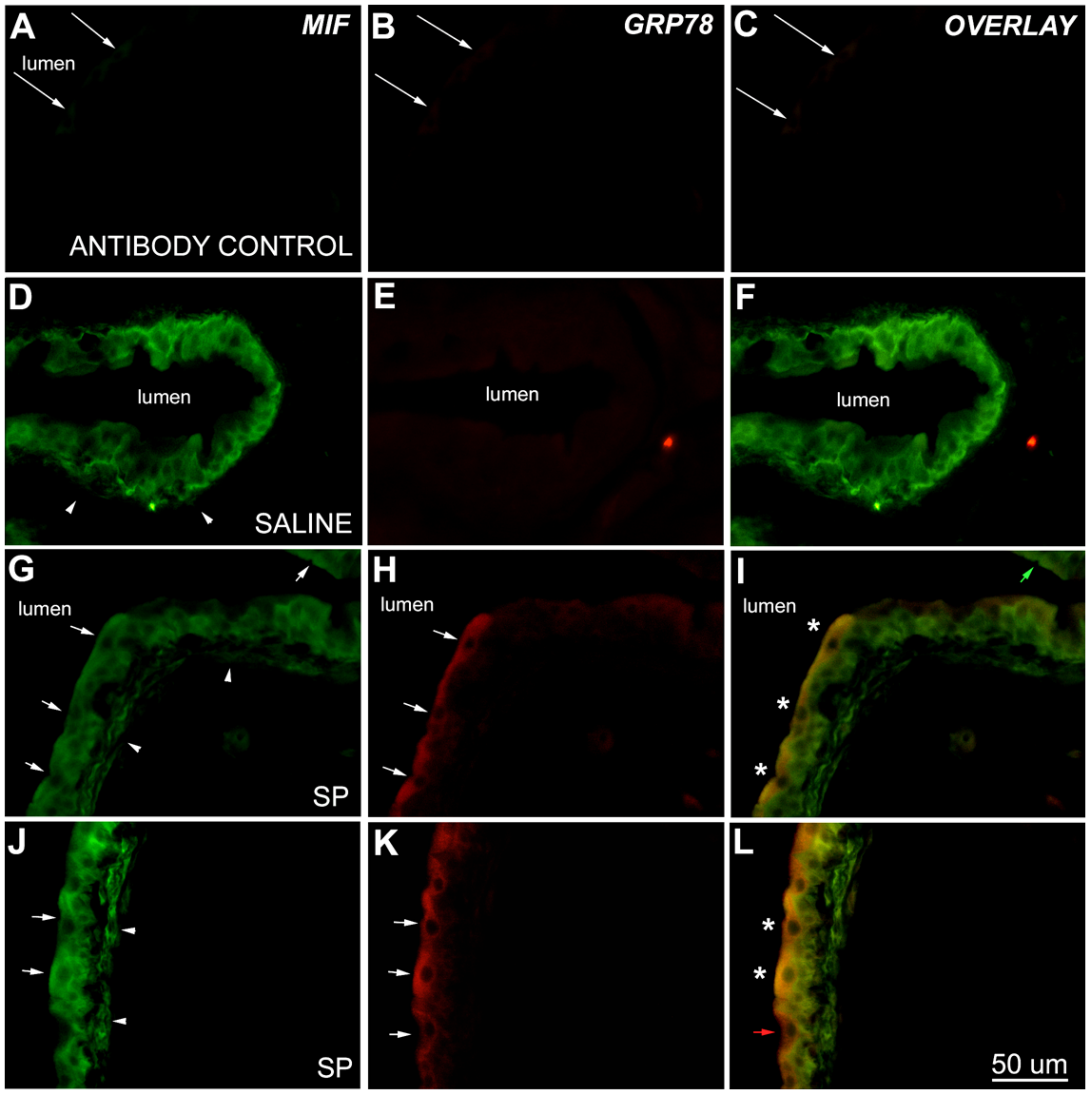
Substance P-induced MIF and GRP78 in umbrella cells. Single panels illustrating MIF (FITC labeling), GRP78 (TRITC labeling) and overlay of both (using Leica Imaging Software). Exposure times were constant for each particular fluorochrome and images were uniformly adjusted for brightness and contrast (Adobe Photoshop Elements v.2; Adobe Systems, San Jose, CA). Control sections where primary antibodies had been omitted showed no MIF (A) or GRP78 immunofluorescence (B). Consequently, overlay panel also shows no immunofluorescence (C). Arrows mark luminal edge of urothelium and slight autofluorescence can be seen. Representative sections showing urothelial immunostaining in saline-treated (D–F) and substance P-treated (G–L) bladders. Saline treated bladders showed MIF (D) immunostaining in the urothelium, whereas the urothelium appeared devoid of GRP78 immunostaining (E). Note MIF staining in basal and intermediate cells and light (or non-existent) immunostaining in umbrella cells (D). Overlay panel shows only MIF immunostaining (F). Arrowheads show location of the lamina propria, showing MIF immunostaining (D). In substance P-treated bladders, on the other hand, both MIF (G,J) and GRP78 (H,K) immunostaining was readily seen in the urothelium, particularly in umbrella cells (arrows). Overlay shows simultaneous MIF and GRP78 immunostaining as an orange color (indicated by asterisks; I,L). MIF only (panel I; green arrow) and GRP78 only (panel L; red arrow) umbrella cells can also be seen in the overlay panels. When compared to saline-treated bladders (D) MIF immunostaining in substance P-treated bladders is also increased in the fibroblast layer of the lamina propria (arrowheads, G,J). Calibration bar = 50 µm.

Bladder sections from saline and SP-treated rats were simultaneously exposed to MIF and GRP78 antibodies, visualized using dual immunofluorescence and single fluorophore images were overlaid. Sections where both primary antibodies had been omitted showed no immunofluorescence (Fig. 1A–C; arrows indicate luminal edge of urothelium). Similarly, omission of either one of the secondary antibodies resulted in only the appropriate fluorescence being observed (not shown). In saline treated rats, MIF immunostaining was observed in basal and intermediate layers of the urothelium as previously described (Fig 1D)[10]; while no GRP78 immunostaining was observed in the urothelium (Fig. 1E) and thus the overlay (Fig. 1F) showed only MIF immunostaining.

After SP treatment, on the other hand, both MIF (Fig. 1G;J) and GRP78 (Fig. 1H;K) immunostaining were observed in the urothelium. Umbrella cells, in particular, showed both (rendered orange color after overlay and marked by asterisks in Fig 1I;L). Umbrella cells containing only MIF (Fig 1I; green arrow) or only GRP78 (Fig 1L; red arrow) were also visible. Intermediate and basal cells were only MIF-positive (Fig 1I;L). In addition, the lamina propia, where only MIF immunostaining was present, also showed increased MIF immunostaining after SP treatment (Fig. 1G;J arrowheads) compared to saline treatment.

### Substance P-induced increase in bladder GRP78-MIF complexes

**Figure 2:**
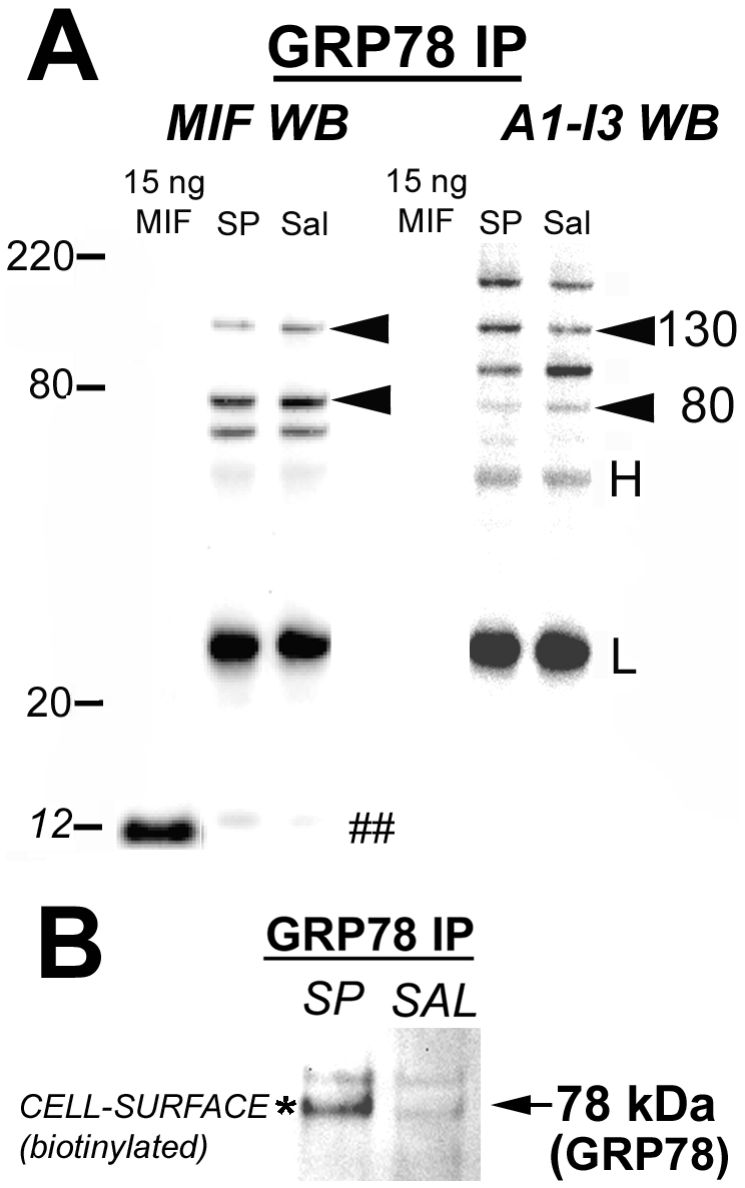
GRP78-MIF complexes in the bladder. A) Representative results of GRP78 “pull-down” experiments where GRP78 complexes were isolated from bladder homogenates by immunoprecipitation and then MIF (left side of figure) or A1-I3 (right side of figure) western blotting was carried out to identify complexes. Recombinant MIF was loaded (1^st^ lane) as positive control. Note that GRP78-MIF complexes (marked by ## at 12 kDa, lane marked Sal = Saline) increased after substance P treatment (lane labeled SP). A1-I3 western blotting (right side) showed several fragments associated with GRP78, as expected, since GRP78 is a receptor for A1-I3. Also note that while 12 kDa MIF was not detected, several high-molecular weight fragments contained both MIF and A1-I3 (130 and 80 kDa), as reported previously[9]. B) GRP78-MIF complexes at the urothelial cell-surface. Urothelial cell-surface proteins were biotinylated, isolated and then GRP78 biotinylated complexes were “pulled down” using GRP78 antibodies. The resultant protein complexes were then electrophoresed and biotin activity was detected using biotin-horseradish peroxidase-chemiluminescence. A faint band of biotinylated (cell-surface) proteins at approximately 78 kDa (corresponding to GRP78 molecular weight) were “pulled down” by GRP78 immunoprecipitation in saline animals, while this band increased in intensity in substance P-treated animals (asterisk). Therefore, these results show that GRP78-MIF urothelial cell-surface complexes increase after substance P-treatment.

We examined GRP78-MIF complexes in the bladder by “pulling down” GRP78 complexes in bladder homogenates, electrophoresing the resultant proteins and performing western blotting for MIF or for A1-I3. Figure 2A is representative of the co-immunoprecipitation studies and shows a representative experiment where the resultant proteins from the same samples were assayed with MIF western blotting (left side of figure) or A1-I3 western blotting (right side of the figure).

GRP78 co-immunoprecipitation of bladder homogenates followed by MIF western-blotting showed a faint band at approximately 12 kDa corresponding to MIF (Fig 2A; left side; lane labeled Sal; marked by ##) after saline treatment and a band of greater intensity is observed after SP treatment (Fig 2A; left side; lane labeled SP). Recombinant MIF is included as a positive control. Also, GRP78 co-immunoprecipitation of bladder homogenates followed by A1-I3 western blotting (right hand Fig 2A) showed several A1-I3 bands (as expected since GRP78 is a receptor for A1-I3) at different molecular weights (200, 130 and 80 kDa) indicating full-length and proteolytic fragments of A1-I3, as reported before [9]. Absence of bands at 12 kDa demonstrates that A1-I3 antibodies do not cross-react with MIF. Bands at 130 and 80 kDa could be seen in both MIF and A1-I3 western blots indicating MIF-A1-I3 complexes at these molecular weights as previously described[9].

### Substance P-induced Cell-surface expression of GRP78

Proteins expressed on the surface of urothelial cells were detected by intraluminal in vivo biotinylation, a method we described in detail recently[11]. After exposure of urothelial cells to biotinylation reagent and collection of urothelial cells (by scraping), the cells were homogenized and biotinylated proteins (i.e. cell-surface proteins) were collected in an avidin column. GRP78 co-immunoprecipitation of such biotinylated proteins, electrophoresis and exposure to biotin-horseradish peroxidase-chemiluminescence revealed the presence of biotinylated (thus located on the surface) GRP78. Lack of vimentin in the samples (not shown) indicated that only urothelial cells and no lamina propria fibroblasts were collected. In addition, our previous experiment showed that only the superficial layer of the urothelium is biotinylated[11]. Figure 2B shows representative results of a biotinylation experiment. A faint band showing biotinylation is present at 78 kDa (corresponding to GRP78, indicated by arrow) in saline-treated bladders whereas SP treatment resulted in a band of greater intensity (asterisk).

### Intraluminal antibodies to CD74 or GRP78 alone had no effect

All of the different experimental groups and intraluminal treatments are listed in Table 1. We examined possible non-specific effects of intraluminal antibody (ab) treatment in rats treated with saline alone. Table 2 shows levels of urothelial MIF, released (intraluminal) MIF and bladder score in 3 controls groups: 1) Saline (subcutaneous; s.c.)+Sal (intraluminally); 2) Saline (s.c.)+CD74 ab (intraluminally) and 3) Saline (s.c.)+anti-GRP78 ab (intraluminally). Intraluminal antibodies to CD74 or GRP78 did not result in significant changes in any of these three measures when compared to saline treatment, as determined by Analysis of Variance (ANOVA; Table 2). Therefore, data from controls groups were pooled for further analysis.

**Table 1:** Experimental design for different intraluminal treatments and their effects on substance P-induced bladder inflammation.

Experimental Groups	Treatment (s.c.)	Treatment (intraluminal)
N = 5	Substance P (40 ug/kg)	Saline (0.3 ml)
N = 5	Substance P (40 ug/kg)	CD74 ab (15 µg/kg; 0.3 ml)^1^
N = 5	Substance P (40 ug/kg)	GRP78 ab(15 µg/kg; 0.3 ml)^2^
N = 5	Substance P (40 ug/kg)	CD74 ab+GRP78 ab (5 µg/kg; 0.3 ml)
N = 5	Substance P (40 ug/kg)	IgG (15 µg/kg; 0.3 ml)^3^
**Control Groups**		
N = 5	Saline	Saline (0.3 ml)
N = 5	Saline	CD74 ab (15 µg/kg; 0.3 ml)^1^
N = 5	Saline	GRP78 ab (15 µg/kg; 0.3 ml)^2^

1 Goat polyclonal; Santa Cruz; sc-5438.

2 Goat polyclonal; Santa Cruz; sc-1050.

3 Goat non-specific IgG; Santa Cruz; sc-2028.

All antibodies were dialyzed to remove sodium azide. Final protein concentrations were determined from all samples (BCA, Thermo Scientific).

**Table 2:** Mean and S.E.M for each parameter in control groups (N = 5/group).

	Sal(Sal)	Sal(CD74)	Sal(GRP)	ANOVA
**Urothelial MIF (ng MIF/mg prot)**	39.98 (±1.55)	44.98(±8.0)	41.34(±4.04)	F = 0.789 n.s.
**Released(Intraluminal) MIF (ng MIF/ml)**	4.04(±0.65)	4.00(±0.87)	3.47(±0.99)	F = 0.865 n.s.
**Bladder Score**	1(±0.32)	0.2(±0.2)	1(±0.32)	F = 0.110 n.s.

Abbreviations: Sal = Saline; CD74 = CD74 antibody; GRP = GRP78 antibody.

### Intraluminal antibodies to CD74 and/or GRP78 prevented substance P-induced urothelial MIF release

**Figure 3:**
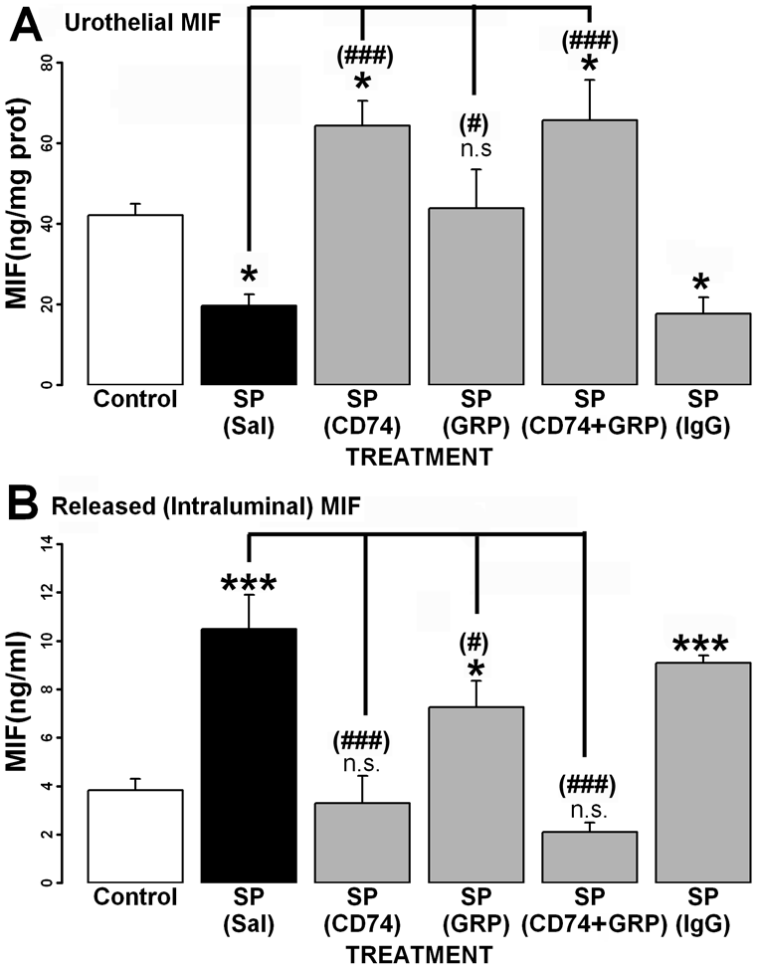
Effect of different intraluminal treatments on substance P (SP)-induced urothelial MIF release. Intraluminal treatment for each group is in parenthesis. A) Urothelial MIF is decreased after SP treatment, suggesting MIF release from the urothelium. This effect is prevented by intraluminal treatment with CD74 ab (CD74), GRP78 ab (GRP) or a combination of both. Intraluminal treatment with non-specific IgG did not prevent the decrease in urothelial MIF produced by SP. B) SP treatment significantly increased MIF in intraluminal fluid. Intraluminal treatment with CD74 ab prevented this increase, whereas treatment with GRP-78 ab (GRP) decreased the amount of MIF in the intraluminal fluid (compared to SP only), but it was still increased (compared to saline). Treatment with both CD74 ab and GRP78 ab (CD74+GRP) also prevented SP-induced MIF release. Intraluminal non-specific IgG had no effect. * = comparison to saline p<0.05; *** = p<0.001; (#) = comparison to SP(Sal) p<0.05;(###) = p<0.001; n.s. = not significant.

Substance P decreased MIF levels in urothelial cells compared to controls (Fig 3A) indicating MIF release from these cells into the intraluminal fluid. Intraluminal CD74 ab and/or GRP78 ab prevented SP's effects, whereas intraluminal non-specific immunoglobulin G (IgG) was not effective. Intraluminal CD74 ab or CD74 ab+GRP78 ab caused an increase in urothelial MIF, when compared to controls.

Concomitantly with decreased urothelial MIF levels, SP treatment significantly increased intraluminal (released) MIF compared to controls (Fig 3B). Intraluminal CD74 ab and CD74 ab+GRP78 ab prevented this increase while intraluminal GRP78 ab alone reduced (but did not prevent) intraluminal MIF release.

### Intraluminal antibodies to CD74 and GRP78 reduced substance P-induced histological changes

**Figure 4:**
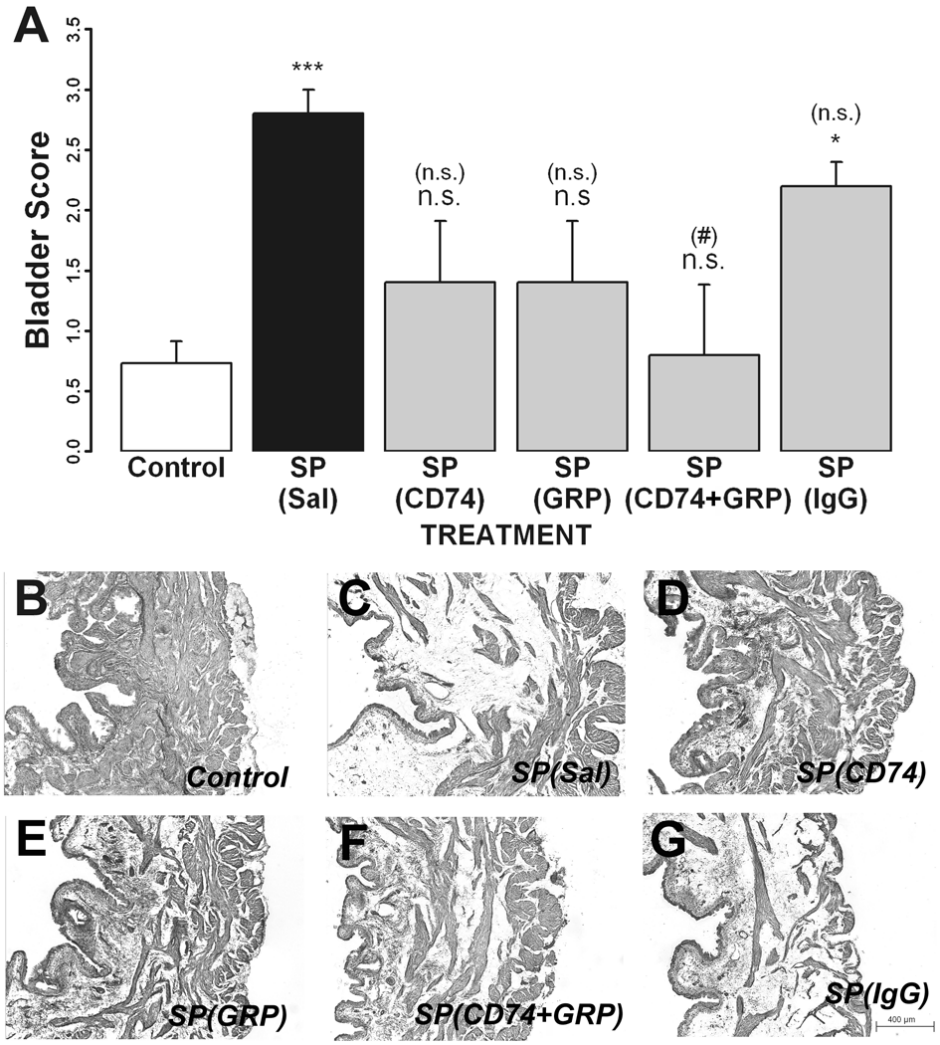
Effect of different intraluminal treatments on substance P (SP)-induced histological changes. A) Bladder inflammation scores across all treatment groups (intraluminal treatment for each group is in parenthesis). Substance P produced bladder edema and inflammation when compared to the control group. Groups treated with CD74 ab (CD74) and/or GRP78 ab (GRP) had bladder scores that were not significantly different from control. B–G. Representative H&E stained bladder sections from each group. * = comparison to saline p<0.05; *** = p<0.001; (#) = comparison to SP(Sal) p<0.05;(###) = p<0.001; n.s. = not significant.

Bladder sections from all groups were stained with H&E and evaluated by an observer (blinded as to the treatment) for histological changes. SP treatment produced sub-mucosal edema and vascular congestion resulting in a higher bladder score compared to control groups (Fig 4A;C). Intraluminal CD74 ab (Fig 4A;D) or GRP78 ab (Fig 4A;E) reduced bladder scores (not significantly different from controls) and a combination of antibodies (CD74+GRP78; Fig. 4A;F) significantly decreased bladder scores compared to SP. Intraluminal IgG had no effect on SP-induced changes (Fig 4A;G). Representative bladder sections from each of the treatment groups are presented in Fig 4B–G.

### Intraluminal antibodies to CD74 or GRP78 reduced substance P-induced expression of inflammatory markers in urothelium

Substance P treatment significantly upregulated (>10-fold change; p<.001) many inflammatory markers in urothelial cells as detected by RT-PCR array, including MIF, INF-γ, TNF-α, IL-1β and CXCL-2 (Table 3A). Table 3A presents changes in SP-induced expression of all markers studied as a function of intraluminal treatment. All expression is normalized to expression in the control group (Saline (s.c.)/Saline (intraluminally). SP, in animals that received only intraluminal saline as a treatment, significantly upregulated most of the inflammatory markers studied (Table 3A). However, intraluminal CD74 ab prevented or reduced expression of inflammatory markers. Similarly, treatment with intraluminal GRP78 ab was effective in preventing or reducing SP-induced expression. However, GRP78 ab also produced increased expression (compared to SP/Saline treatment) in certain markers (denoted with asterisk). Therefore, the effects of CD74 ab and GRP78 ab were not identical.

**Table 3:** Changes in substance P-induced expression of inflammatory markers in urothelial cells as a result intraluminal treatment.

		Intravesical Treatment				Intravesical Treatment		
		SALINE	CD74	GRP78		SALINE	CD74	GRP78
**A. UPREGULATED**								
	***Chemokines***				***Cytokines***			
	Ccl17	***7140***	***29***	14	Ifng	***19032***	***519***	**33199** ^*^
	Il13	***6671***	37	2	Il11	***18828***	***749***	1
	Cxcl5	***4211***	***60***	−4	Il18	***15857***	***324***	**2422**
	Ccl19	***3433***	6	−3	Il1f6_predicted	***15215***	***34***	**102360** ^*^
	Cxcl10	***3377***	8	***18806*** ^*^	Il5	***14053***	−1	**1768**
	Cxcl11	***3121***	474	6	Il17b	***9607***	***64***	14
	Ccl11	***2825***	***387***	−1	Tnf	***9240***	***23***	**169**
	Cxcl2	***2697***	2	***410***	Itgam	***6360***	8	−2
	Ccl7	***1630***	13	4	Tnfsf5	***4280***	2	**4683** ^*^
	Ccl2	***1447***	10	502	Il3	***4224***	***501***	−2
	Ccl9	***1311***	−2	−7	Il1a	***2503***	***56***	82
	Ccl24	***1225***	5	1	Il1b	***2372***	***109***	−7
	Ccl20	***851***	***95***	−2	Il16_mapped	***2179***	5	−7
	Ccl21b	***813***	***19***	***313***	Il4	***2162***	***57***	**1379**
	Ccl3	***619***	−16	−14	Spp1	***1684***	1	−5
	Cxcl1	***580***	1	1	Lta	***1456***	35	26
	Cxcl12	***122***	26	−63	Il10	***1133***	***32***	2
	Pf4	***37***	−2	***467*** ^*^	Itgb2	***647***	−8	−26
	Ccl5	***24***	−3	−21	Il1f5_predicted	***194***	−2	1
	***Chemokine Receptors***				Ltb	***179***	12	5
	Cx3cr1	***20715***	386	1	Mif	***149***	***34***	−***102***
	Il8rb	***10866***	***41***	−2	Scye1	***70***	−***281***	−***116***
	Ccr8_predicted	***7424***	−3	−1	***Cytokine Receptors***			
	Il8ra	***5687***	−1	−4	Il1r2	***13245***	***109***	−2
	Xcr1_predicted	***5192***	***68***	−2	Il5ra	***11039***	***327***	***2549***
	Ccr4	***3881***	110	***808***	Il2rb	***6345***	***38***	2
	Ccr3	***2207***	−10	−6	Tnfrsf1a	***3585***	5	−4
	Ccr7	***2056***	−11	***15514*** ^*^	Il13ra1	***2894***	***74***	−3
	Ccr2	***1835***	***16***	3	Il2rg	***1217***	***167***	**52**
	Ccr6	***1714***	***28***	***23457*** ^*^	Tnfrsf1b	***620***	11	−31
	Ccr5	***857***	2	−15	Il10ra	***142***	***6***	**55**
	Cxcr3	***707***	14	**9448** ^*^	Il1r1	***69***	−9	5
	Ccr9	***389***	−1	**4888** ^*^	Il6r	***20***	−1	−2
	Gpr2_predicted	***261***	4	3	Il6st	***19***	−2	−***579***
					***Others***			
					Crp	***14553***	***659***	***160***
					C3	***3366***	***55***	6
**B. UNCHANGED**								
	***Chemokines***				***Chemokine Receptors***			
	Ccl12_predicted	162	1	−28	Ccr1	763	62	−7
	C5	60	2	1	***Cytokines***			
	Ccl6	29	−19	−22	Tgfb1	6	−2	17
	Ccl4	27	1	12	***Others***			
	Ccl22	27	2	3	Casp1	85	−15	4
	Ccl25	20	−7	−103	Tollip_predicted	15	−***79***	−***1178***
	Cx3cl1	9	−3	−1	Bcl6_predicted	5	−80	255
	Cxcl9	−1	3	3	Blr1	−37	−40	−5

Expression is calculated as fold-change over control treatment, Saline (s.c.)/Saline (intraluminal). Significant changes (highlighted in italicized bold) were defined as >10-fold expression and with p<0.001. Asterisks after individual markers in GRP78 ab treatment group denote increased SP-induced expression over Saline treatment that differed from CD74 ab.

**Figure 5:**
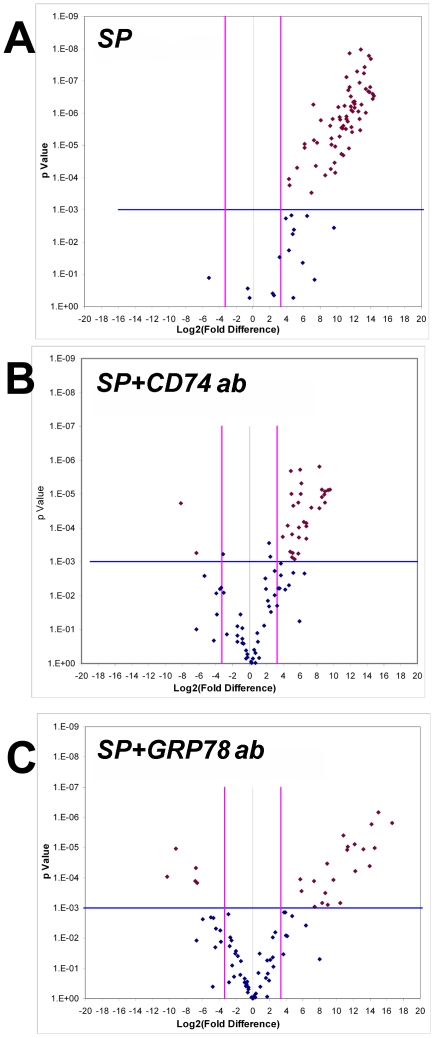
Volcano Plots of inflammatory gene array changes. For each plot, fold change in expression for each of the inflammatory cytokines studied (Table 2) is plotted on the abscissa while t-test significance is plotted on the y-axis. In all three plots, comparison of gene expression is made to control group (Sal (s.c.)/Sal(intraluminal). A significant change was arbitrarily defined as a 10-fold difference (vertical lines) AND a p<0.001 (horizontal line). Using these criteria then, significant changes have been plotted using red markers, while non-significant changes are plotted using blue markers. A) Substance P treatment with saline as an intraluminal treatment (SP = Substance P(s.c.)/Saline (intraluminal) upregulated many inflammatory genes (red points in upper right quadrant). B) Intraluminal CD74 ab treatment, in SP treated rats (SP+CD74) reduced the number of inflammatory genes upregulated by SP. C) Similarly, intraluminal GRP78 ab treatment also reduced the number of genes upregulated by SP with some showing significant downregulation (red points in upper left quadrant).

The information in table 3 is also presented in the form of a volcano plot[20]. Figure 5A shows upregulated markers in SP treated (intraluminal saline) compared to saline treatment. Intraluminal CD74 ab or GRP78 ab (Fig 5B;C) markedly reduced the number of inflammatory markers upregulated, with some showing significant downregulation. Finally, table 3B lists markers that were not upregulated (<10 fold and/or p<0.001) by SP treatment.

## Discussion

In the present study we show that SP treatment induced expression of GRP78 in umbrella cells, which also showed MIF immunostaining. Moreover, GRP78-MIF and GRP78-A1-I3 complexes were detected from bladder homogenates. Finally, in vivo biotinylation experiments showed that SP induced GRP78 expression in the cell surface of urothelial cells. Cell-surface expression of GRP78 receptors in epithelial cells from pelvic viscera has been reported[21]–[23] and mediates signal transduction[21]. Thus, our present findings, novel for the bladder during inflammation, confirm other reports for pelvic viscera.

Our results also show MIF is released specifically from urothelial cells during inflammation. Earlier work had shown constitutive MIF expression in urothelial cells[10], decreased bladder homogenate MIF and increased intraluminal (released) MIF[3], [5] and upregulated MIF mRNA in urothelial cells[11]. Examining isolated urothelial cells (this study), demonstrated that treatment with SP decreases MIF levels in these cells, while increasing released MIF. Therefore, while not ruling out a contribution from the rest of bladder to MIF release, our evidence clearly shows that the urothelium contributes significantly to MIF release during inflammation. Released MIF and MIF-A1-I3 complexes are available to bind to urothelial (cell-surface) CD74 and GRP78 and possibly activate a pro-inflammatory cascade in the bladder. Therefore, the function of these receptors during bladder inflammation was examined using intraluminal antibodies to both receptors.

In support of our hypothesis, intraluminal antibodies to CD74 and/or GRP78 prevented SP induced changes, namely: 1) MIF release from urothelial cells 2) reduced or prevented histological bladder changes and 3) decreased expression of inflammatory markers in urothelial cells induced by SP. Given that intraluminal non-specific IgG was not effective in preventing SP-induced changes and also that antibodies to CD74 or GRP78 did not, by themselves, alter bladder parameters measured in this study, we conclude that the effects of antibodies to CD74 and GRP78 were specific to MIF-receptor interactions. Interestingly, intraluminal antibodies to CD74 (either alone or in combination with GRP78) increased urothelial MIF content suggesting that activation of CD74 regulates MIF release. We also recently observed in vitro that reducing CD74 mRNA (using RNA interference) upregulated MIF in prostate cancer cells [24]. While it is possible that CD74 may act as cargo molecule for MIF and thus modulate MIF release from urothelial cells, this possibility remains to be tested. However, we recently provided evidence that substance P induced urothelial surface expression of CD74[11]. Therefore, it is possible that activation of urothelial cell-surface CD74 (MIF's receptor) but not GRP78 (MIF-A1-I3 complex receptor) modulates MIF's release from the cell and MIF production inside the cell, with receptor down-regulation (or blockade) increasing MIF expression. Regulating MIF expression may thus play a role in inflammation (present results) and carcinogenesis[24].

Intraluminal antibodies to MIF prevented or reduced SP-induced inflammatory changes in the bladder[5]. Our present results extend those findings since intraluminal antibodies to CD74 (receptor for MIF) and/or antibodies to GRP78 (receptor for MIF-A1-I3 complexes) were also effective in reducing of preventing inflammatory effects of SP in the bladder. Taken together, our results show that preventing MIF from binding to specific urothelial receptors reduces or prevents cystitis. Although the present study focused on CD74 (canonical MIF receptor) and described a novel MIF-complex receptor (GRP78), it is likely that other urothelial receptors may bind MIF and thus participate in MIF-mediated signaling in the bladder. CXCR2 and CXCR4 have recently been described as non-canonical MIF receptors[14], [25], and CXCR4 was recently shown to be located in the normal urothelium, to bind to MIF and to be upregulated during bladder inflammation[15]. CXCR2 is also expressed by urothelial cells[26] and may also be involved in this process. Therefore, future investigations into the specific role of these urothelial receptors in MIF-mediated signal transduction in the bladder should increase our understanding of MIF-mediated signaling during bladder inflammation.

### Conclusions

Constitutively expressed urothelial MIF is released by SP and participates in bladder inflammation (either maintaining or enhancing inflammation) by interacting with luminal cell-surface receptors CD74 and GRP78.

## Materials and Methods

### Ethics Statement

All experiments were conducted after obtaining Institutional Animal Care and Use committee approval (protocol #2643) and conformed to the National Institutes of Health Guide for animal experimentation.

### Substance P-induced bladder inflammation

In anesthetized rats (male Sprague-Dawley; 300–350 gm; Harlan, IN; sodium pentobarbital; 60 mg/kg; i.p.), the bladder was exposed by abdominal incision and ureters were cut to isolate the bladder. This allowed for control of the type and amount of intraluminal fluid. Urine was removed from the bladder using a syringe (30 ga needle) and replaced with 0.3 ml of saline. Then rats received either saline (0.1 ml/100 g body weight; s.c.; N = 5) or SP (Sigma; dissolved in saline; 40 µg/kg; 0.1 ml/100 g body weight; s.c.; N = 5)[3]–[5], [7], [9], [16]. After 1 hour the intraluminal fluid was collected and the bladder was excised and bisected (longitudinally). Bladder halves were either placed in 4% paraformaldehyde or processed for protein and/or mRNA.

### MIF and GRP78 dual immunofluorescence

Frozen, coronal bladder sections (12 µm) from rats treated with saline or SP were exposed simultaneously to MIF (1∶200; rabbit polyclonal; Torrey Pines Biolabs; Houston, TX) and GRP78 (1∶200; goat polyclonal; sc-1050; Santa Cruz Biotechnology; Santa Cruz, CA) antibodies and visualized using appropriate secondary antibodies conjugated to fluorescein isothiocyanate (FITC) or tetramethylrhodamine isothiocyanate (TRITC; Jackson Immunochemical; West Grove, PA). Primary or either secondary antibodies were omitted in control sections. Slides were examined using a Leica DMI4000B (Leica Microsystems Inc, Wetzlar, Germany) microscope.

### GRP78-MIF or GRP78-A1-I3 complex detection using co-immunoprecipitation followed by Western blotting

GRP78 protein complexes were isolated from rat bladder homogenates (250 µg protein) by first immunoprecipitating using GRP78 polyclonal antibody (5 µg) and protein G agarose. Resultant protein complexes were separated by sodium dodecyl sulfate–polyacrylamide gel electrophoresis (SDS-PAGE) and transferred to polyvinylidene difluoride (PVDF) membranes. GRP78-MIF or GRP78-A1-I3 complexes were detected using using Western blotting with either MIF biotinylated goat polyclonal antibody (R&D Systems; Minneapolis, MN, BAF289) or A1-I3 rabbit polyclonal antibody (a gift from Professor Harry van Goor), streptavidin-horseradish peroxidase conjugate and chemiluminescent substrate (Pierce, Rockford, IL). Bands were visualized and quantified using Kodak Image Station (Kodak, Rochester, NY) and included software.

### *In vivo* biotinylation of luminal cell-surface proteins

We used in vivo biotinylation of luminal cell-surface proteins as we described recently[11]. Briefly, rats were divided into saline (N = 5) or SP (N = 5) groups as described above. Bladders were emptied of urine, rinsed twice with PBS and filled with biotinylation reagent (1 mg/ml N-hydroxysulfosuccinimide biotin ester, Pierce Biochemicals, Rockford, IL; 0.3 ml). After 1 hour the bladders were excised, carefully stretched and urothelial cells collected by scraping (epithelial aggregate separation and isolation; EASI)[27]. Remaining bladder pieces were fixed in formalin and frozen sections stained with hematoxylin and eosin (H&E) to document that only epithelium was removed. Also, western-blotting using anti-vimentin (a fibroblast marker) was used to verity that only urothelial cells were isolated[11]. Isolated urothelial cells were collected by centrifugation at 10,000 g (5 min), washed (3x) in PBS pH 8.0 to inactivate residual biotinylation reagent. Cells were lysed with SDS and homogenates were immunoprecipitated with anti-GRP78 antibodies (described above). After electrophoresis and transfer to PDVF membranes, biotinylated surface proteins were visualized using only chemiluminescent substrate in the Kodak Image Station.

### Effects of intraluminal antibodies to GRP78 and/or CD74 on SP-induced urothelial MIF release, bladder histological changes and urothelial expression of inflammatory markers

Table 1 lists intraluminal antibody treatments in experimental and separate control groups (testing for possible effects of intraluminal treatment alone in the absence of SP). Saline (s.c.) or SP (s.c.) administration to the groups was as described above. One hr after SP treatment, intraluminal fluid was collected and bladders excised. Urothelial cells were collected from half of the bladder using the EASI procedure (see above) while the other half of the bladder (intact) was placed in paraformaldehyde. Intraluminal fluid and urothelial cell homogenates were processed for MIF protein using enzyme-linked immunosorbent assay (ELISA)[15].

To assess changes in urothelial expression of inflammatory markers, total RNA (1 µg) from scraped urothelial cells from animals in each of the following groups: SP (s.c.)/Saline (intraluminal), SP (s.c.)/Anti-CD74 (intraluminal), SP (s.c.)/Anti-GRP78 (intraluminal) and Saline (s.c.)/Saline (intraluminal) were separately pooled and reverse transcribed (RT^2^ First strand kit, C-03, SABiosciences, Fredrick, MD). The resulting cDNA was used in a PCR array (repeated 3 times; Inflammatory Cytokines and Receptors, PARN-011, SA Bioscience) and analyzed using GEarray Expression Analysis Suite (http://geasuite.superarray.com/; SABiosciences). Gene expression was normalized to ribosomal protein RPL13A. 10-fold gene expression changes (significant at p<0.001) when compared to Saline (s.c.)/Saline (intraluminal) were considered significant.

Bladder histological changes were assessed in frozen bladder sections (12 µm; groups in Table 1) counterstained with H&E and examined by an observer blinded to experimental treatment and received a score of: 0 = no edema, normal appearance; 1 = slight submucosal edema and vascular congestion, but not present uniformly; 2 = moderate submucosal edema and vascular congestion; 3 = severe submucosal edema, vascular congestion, partial loosening of intercellular urothelial junction.

### Data analyses

Data presented are Mean±S.E.M. ANOVA followed by Tukey Honest Significant Difference tests were performed using statistical software (R; http://www.r-project.org/). Gene expression changes were analyzed using GEarray Expression Analysis Suite (http://geasuite.superarray.com/; SABiosciences). A 10-fold gene expression change with a t-test showing p<0.001 was considered significant. The effect of intraluminal treatment on SP-induced inflammatory gene expression changes were plotted using a volcano plot[20]. A volcano plot identifies significance and magnitude of change in expression of a set of genes between two conditions. It is a scatter plot of the negative log10 transformed p-values from a t test on the y-axis against the log2 fold change between the two conditions on the x-axis. The x-axis indicates biological impact of the change; the y-axis indicates the statistical evidence, or reliability of the change. Genes with statistically significant differential expression according to the gene specific t-test will lie above a horizontal threshold line (set at 10-fold). Genes with large fold-change values will lie outside a pair of vertical threshold lines (set at p<0.001). The significant genes identified by the t-test will tend to be located in the upper left or upper right parts of the volcano plot.
